# The Role of Iron Metabolism in Lung Inflammation and Injury

**DOI:** 10.4172/2155-6121.S4-004

**Published:** 2012-01-25

**Authors:** Jonghan Kim, Marianne Wessling-Resnick

**Affiliations:** Department of Genetics and Complex Diseases, Harvard School of Public Health, Boston, MA 02115, USA

**Keywords:** Iron status, Oxidative stress, Metal exposure, Inhalation, Bronchoalveolar lavage

## Abstract

Iron is required for many vital functions including oxygen transport and energy metabolism. Protective mechanisms maintain optimal iron concentration involving dynamic regulation of the transporters and iron storage proteins. In addition to these systemic regulatory mechanisms, the unique lung environment must provide detoxification from metal-induced oxidative stress and pathogenic infections. This review focuses on the unique role of iron metabolism in lung injury and inflammation.

## Introduction

Iron is required for many vital functions including oxygen transport and energy metabolism. About ¾ of total body iron is present in heme associated with hemoglobin, myoglobin and cytochromes, while nonheme iron is either stored in tissues or transported in the circulation bound to the serum protein transferrin [1]. Low body iron status results in iron-deficient anemia, impaired motor activity and poor brain development [2–5]. On the other hand, high iron stores promote oxidative stress triggering inflammatory responses and cellular injury that eventually leads to cell damage and death. The body has therefore developed protective mechanisms to maintain optimal iron concentration. Protective feedback includes dynamic regulation of the expression of transporters and proteins for iron storage. In addition to these systemic regulatory mechanisms, the unique lung environment must provide detoxification from metal-induced oxidative stress and pathogenic infections. Comprehensive reviews about the molecular mechanisms of iron regulation are available [6–8], therefore we will restrict our focus to the role of iron metabolism in lung injury and inflammation.

## Overview of Iron Metabolism in the Body

The serum concentration of iron at steady-state is closely governed by absorption and clearance of iron. Both heme and non-heme forms of iron are absorbed from the gut, but through different transport mechanisms. Non-heme iron import begins with the reduction of ferric iron (Fe^3+^) by duodenal cytochrome b (Dcytb) protein to ferrous iron (Fe^2+^), which is then taken up by divalent metal transporter 1 (DMT1) at the duodenal apical membrane into the cytosol [9–11]. Less is known about the import of dietary heme, although candidate transporters have emerged [12,13]. Upon entering the intestinal enterocyte, intracellular iron can be incorporated in ferritin for storage or exported across the basolateral surface into circulation by ferroportin (FPN) [14,15]. FPN is assisted by hephaestin, a membrane-bound ferroxidase that converts Fe^2+^ to Fe^3+^ to promote iron binding to transferrin for its delivery to peripheral tissues [16].

Transferrin (Tf) delivers iron to erythroid cells where DMT1 appears also to be essential for iron acquisition necessary for heme production [9,17]. Red blood cells contain the most abundant pool of iron in the body in the hemoglobin complex. After destruction of senescent erythrocytes, non-heme iron is stored within macrophages or returns to the circulation where iron is recycled for another round of erythropoiesis. There is no known pathway for iron excretion. Excess iron is stored in the liver and other parenchymal cells, typically in the ferritin-associated form. Since iron is efficiently conserved, body iron stores are mainly controlled by regulation of iron absorption [6,18].

The absorption of iron is regulated transcriptionally, post-transcriptionally and post-translationally. The two major iron transporters, DMT1 and FPN, are negatively regulated by body iron status at the transcriptional level [19,20]. FPN transcripts and several DMT1 splice variants also contain Iron-Responsive Elements (IREs) that confer post-transcriptional regulation by Iron-Responsive Proteins (IRPs). In addition, the liver senses iron status through a series of molecular events to promote the release of hepcidin into the blood. Hepcidin is a 25-amino acid polypeptide hormone and plays a central role in iron homeostasis by responding not only to the body iron status but also to inflammation and hypoxia. Hence, the body iron level and plasma hepcidin concentration are positively related [21,22]. Hepcidin binds to FPN and facilitates its degradation, thereby reducing iron absorption [23]. Recent evidence hints at hepcidin regulation of DMT1 by a degradative pathway as well [24]. Evolution has presumably found two pathways to protect the body from iron toxicity: a) regulation of absorption by controlling transporters via hepcidin and b) storage to detoxify excess iron by sequestering in a less reactive form.

### Iron homeostasis in the lung

In the lung, iron distributes in both extracellular and intracellular fluids. After intratracheal instillation of ^59^Fe in rats, radioisotope is found largely in lung tissues (54%) with remaining metal associated with bronchoalveolar lavage (BAL) protein as bound (22%) and unbound (5%) forms [25]. BAL cell pellets (1%) also contain iron, but this value might be underestimated because the recovery of BAL cells depends on the efficiency of lavage. Notably, systemic iron-deficiency with low serum and liver iron concentrations does not seem to affect lung iron levels [26], whereas iron overload is associated with high iron levels in the lung [27]. In addition, pulmonary epithelial lining fluids contain a variety of antioxidant molecules, including mucin, ascorbate, reduced glutathione and other proteins, which not only protect the lungs from oxidative stress [28], but also alter iron’s redox potential and bioavailability.

**Figure 1:**
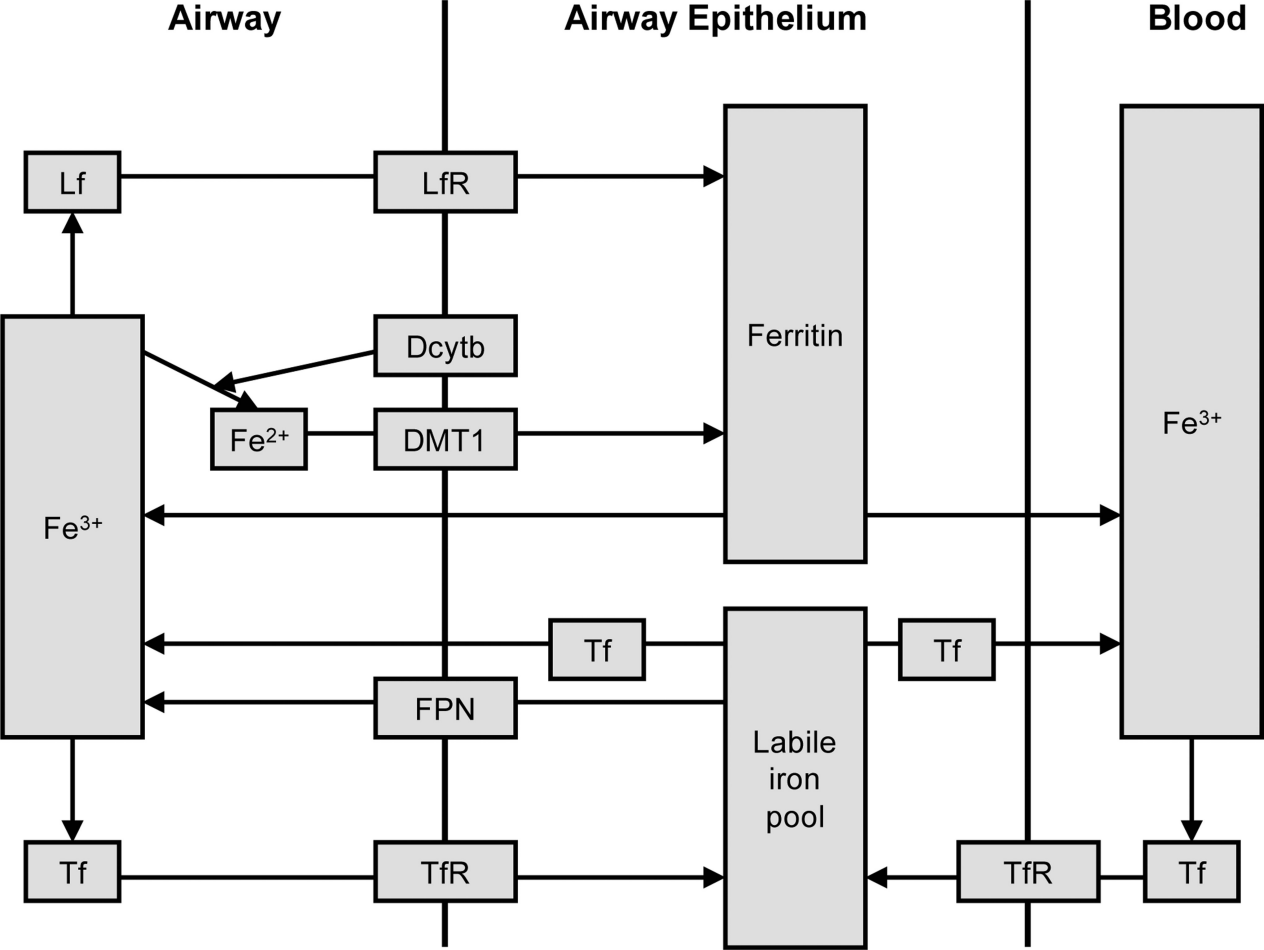
Iron homeostasis in the lung epithelium Shown is a model of iron transport and homeostasis. Abbreviations used: Dcytb: Duodenal cytochrome b; DMT1: Divalent Metal Transporter 1; FPN: Ferroportin; Lf: Lactoferrin; LfR: Lactoferrin receptor; Tf: Transferrin; TfR: Transferrin receptor.

Lung epithelial cells express a variety of iron-associated molecules that serve specific functions (Table 1). These include transferrin (Tf) and its receptor (TfR), lactoferrin (Lf) and its receptor (LfR), ferritin, DMT1 and FPN. Macrophages express natural resistance-associated macrophage protein-1 (Nramp1) as well as most of the molecules that are expressed in the epithelial cells, while neutrophils participate in iron homeostasis by releasing several cell-specific modulators, including Lf, superoxide anion (O_2_-) and siderocalin. Pulmonary uptake of airborne particulate matter accounts for ingestion of 10 µg iron every day [29,30]. Although inhalation is not a major pathway of iron acquisition, continuous exposure to elevated levels of iron may result in greater risk of metal-related toxicity. Multiple cells participate in lung iron homeostasis, as reviewed in reference [38]. Figure 1 focuses on airway epithelium and diagrams the functions of iron-associated molecules listed in Table 1. Their functions are detailed below.

### Lung iron metabolism and infection

Iron metabolism is intrinsically linked to innate immunity by regulation of iron availability to pathogens. Infection and inflammation lead to lung injury. A considerable body of epidemiological evidence indicates that iron stores are associated with disease susceptibility and inflammatory responses. High iron status is related to many infectious diseases and inflammatory responses, as exemplified by malaria, viral infection and neurodegeneration. Detailed information about iron and systemic infection-inflammation can be found in several recent reviews [8,31]. For example, iron administration is known to increase mycobacterial growth [32,33], resulting in increased morbidity and mortality [34,35] and dietary iron is associated with occurrence and death from tuberculosis [36]. On the other hand, iron deficiency appears to provide a protective mechanism from infection by limiting iron utilization [37] and by improving the inflammatory condition [8].

Among the factors listed in Table 1, lung Tf not only imports iron from the airway into the epithelial cells but it also is involved in iron transport from the intracellular space to the outside of the cell, either blood or the airway. Unlike serum levels, pulmonary Tf is unchanged upon iron exposure [25]. Transcripts for its receptor are detected in bronchial epithelium, type II alveolar cells, macrophages and bronchus-associated lymphoid tissue. Since Tf expression is not influenced by systemic iron overload, a local regulation of Tf-TfR expression is postulated [25]. The expression of TfR diminishes in response to inflammation, efficiently depleting iron required for the growth of pathogens such as *L. pneumophila, M. tuberculosis* and *M. avium* [39–42]. Lf is another lung iron binding protein found in most surface secretions that also modifies iron availability. Airway iron binds to Lf and is taken up by LfR present in the lung epithelial cells and macrophages, followed by the storage of iron in ferritin. Lung infection appears to induce Lf release through inflammatory cytokines [31]. However, while exogenous Lf ameliorates pulmonary *M. tuberculosis* in a mouse model of iron overload [43] and opportunistic *P. aeruginosa* infection in patients with cystic fibrosis [44,45], Lf knock-out mice do not exhibit increased susceptibility to these pathogens [46].

Nramp1 has a more defined role in innate immunity as the name “natural resistance” implies. Nramp1 is a divalent metal transporter in phagosomes and reduces phagosomal iron [47] in a pH-dependent fashion and confers resistance to intraphagosomal parasites [47,48]. Infection also increases the expression of Nramp1 and Nramp1 knockout mice are more vulnerable to infection [49]. Nramp1 is also involved in suppression of IL-10 expression by iron mobilization, resulting in elevated iNOS production by macrophages, which restricts microbial growth [50–52].

While Tf significantly contributes to iron transport across the lung epithelium, non-Tf bound iron (NTBI) can also enter the cell through the action of DMT1. DMT1 (also known as Nramp2) is a closely related homolog expressed in the lung [26,53,54]. In rat lungs, DMT1 protein is predominantly found in normal airway and alveolar epithelium, especially type II cells [53], while DMT1 transcripts are also found in bronchus-associate lymphoid tissue adjacent to large airways [53]. Unlike intestinal DMT1, lung DMT1 mRNA does not appear to be highly regulated by iron status [27,53,55]. As a consequence, pulmonary iron absorption is not always correlated with lung iron status [53]. For example, Dcytb, which is found in airway epithelial cells and functions as a ferrireductase to support DMT1 function, is elevated by iron exposure [56]. Furthermore, exposure of the lungs to iron oxide particles can promote up-regulation of DMT1 mRNA in alveolar macrophages and nearby epithelial cells [53]. Hence, DMT1 expression may be more directly altered by extracellular iron levels in the lung rather than by changes in intracellular or systemic iron levels. In addition, proinflammatory cytokines, such as TNF-α and interferon-γ, as well as inorganic fiber like asbestos, promote DMT1 up-regulation in bronchial epithelial cells [57,58]. These data suggest that DMT1 contributes to uptake and detoxification of iron through regulation of non-IRE mRNA isoforms in the lung [57,59]. Indeed, Belgrade rats with DMT1 deficiency display increased pulmonary inflammation in the resting state [60]. Lung inflammation mediated by intratracheal instillation of lipopolysaccharide in Belgrade rats is greater than control rats [60]. Taken together, these observations indicate DMT1 functions to provide a protective barrier from toxic environmental stimuli resulting from airborne metals [59–61].

The hepcidin-FPN axis of regulation provides a more direct link between cellular iron availability and inflammation. This iron-responsive peptide depletes circulating iron by inhibiting FPN function such that iron is unavailable to extracellular pathogens. While high serum iron appears to be associated with several microbial infections [62], reduced cellular iron levels found in hemochromatosis appear to provide resistance to infection, particularly intracellular pathogens. For example, *M. tuberculosis* growth and iron acquisition is significantly impaired in iron-depleted macrophages isolated from patients with HFE-associated hemochromatosis [63]. This mechanism is further supported by other models wherein FPN over-expression leads to impaired growth of intracellular pathogens with the addition of hepcidin enhancing their growth except in cells expressing mutant FPN [64,65]. Hence hepcidin plays an important role in pulmonary inflammation through its regulation of iron transport. In addition, hepcidin was initially identified as liver-expressed antimicrobial peptide (LEAP-1) due to its intrinsic antimicrobial activity and it has been proposed that hepcidin contributes to the innate defense system [66,67]. Interestingly, airway epithelial cells express hepcidin in response to interferon-γ, likely providing a direct protective mechanism against microbial growth [68]. These findings indicate two essential roles of hepcidin: a systemic irondependent interaction with FPN to maintain balance between nutrition and bacterial and viral pathogenicity in lung tissue as well as the body and an iron-independent, direct antimicrobial activity [31].

There is emerging recognition of the role siderocalin plays in iron metabolism. Also known as neutrophil-gelatinase-associated lipocalin or lipocalin-2, it is produced by neutrophil granules and in epithelial cells in response to inflammation. Siderocalin interferes with bacterial iron acquisition to inhibit growth [69] and transports Fe^3+^ in the blood in a complex with endogenous catechol or siderophores secreted by invading pathogens [70]. Pneumonia induced by intratracheal instillation of *E. coli* is exacerbated in siderocalin-deficient mice [71]. Furthermore, infection with active pulmonary tuberculosis in patients appears to be inversely associated with serum levels of siderocalin [72]. Therefore, siderocalin appears to provide a substantial contribution to restriction of iron availability to pathogens and prevention of infection specifically in the lung.

### Lung injury and iron

Altered iron metabolism is linked to many lung diseases [38,73–75]. Elevated iron concentrations in the lung are associated with increased risk of pulmonary injury [38,75–77]. Among many organs in the body, the lung may have the greatest susceptibility to metal-induced oxidative stress due to its unique anatomical role for massive oxygen exchange along with large blood supplies. For example, inhaled iron from occupational settings or sites contaminated by heavy metals may promote reactive oxygen species. Changes in oxygen availability, such as hypoxia and hyperoxia, also alter iron metabolism. Lung injury is characterized by severe hypoxemia, increased endothelial and epithelial permeability, increased cytokine levels in the lungs and neutrophilic alveolar infiltrates [78]. Both acute and chronic lung injury leads to disruption of iron homeostasis in the lung [38]. The relationships between various types of lung injury and the regulation of iron metabolism are discussed below.

Acute respiratory distress syndrome (ARDS) is a type of inflammatory lung injury followed by endothelial activation and disruption of capillary membrane resulting in protein leakage [79,80]. Superoxide and hydrogen peroxide participate in the etiology of ARDS combined with ability of iron to catalyze more toxic reactive oxygen species [81–84]. Hence, iron can exacerbate ARDS [85]. High serum ferritin is associated with the development of ARDS [73]. Ferritin stores iron, distributing between extracellular and intracellular spaces to play a detoxifying role (Table 1, Figure 1). When iron levels increase, ferritin also increases to sequester reactive iron and as an acute reactive protein, ferritin synthesis is elevated during the inflammatory response. Increased ferritin levels observed in ARDS may result from increased tissue damage and lysis [73]. Since chelatable low molecular weight iron in respiratory extracellular fluid becomes elevated in patients with ARDS compared to normal healthy volunteers, it has been proposed that the presence of pro-oxidant iron in lung epithelial fluid may contribute to susceptibility to oxidative damage [28]. Lavage fluid of ARDS patients has elevated levels of total and nonheme iron as well as cellular content of Tf, ferritin and Lf [86]. This indicates impaired pulmonary homeostasis of iron in ARDS, although it is unclear whether this is due to general increase in membrane permeability or altered iron metabolism.

Pulmonary alveolar proteinosis (PAP) is characterized by abnormal accumulation of protein-rich surfactant in the lung and impaired pulmonary functions [87,88]. Patients with primary or idiopathic PAP show increased levels of iron, Tf, TfR, Lf and ferritin in lung lavage fluids, while the concentrations of antioxidants such as ascorbate and glutathione are reduced [74]. Moreover, intracellular concentrations of iron and ferritin are also elevated, suggesting metal-catalyzed oxidative stress [74]. It has been proposed that lower iron saturation of Tf decreases iron-mediated oxidative stress and rescues respiratory failure [89,90]. Secondary PAP can accompany infection, particle exposure and malignancies [38], most of which are associated with altered iron homeostasis. Together, a remarkable relationship between PAP and iron metabolism exists.

Smokers, whether they have bronchitis or not, display elevated iron levels in the lung [91], presumably due to formation of iron complexes by particulate matter in cigarette smoke [38]. Patients with cystic fibrosis also have elevated levels of iron and iron-related proteins in fibrosis also have elevated levels of iron and iron-related proteins in the lavage fluid and sputum compared with normal humans or patients with chronic obstructive pulmonary disease [92,93]. The elevated iron levels in cystic fibrosis are higher than those found in smokers [93] and are associated with increased inflammatory cytokines [92], indicating the significant role of iron in promoting oxidative injury in the lung [92]. Iron also modifies oxidative injury with ischemia/ reperfusion in the lung since released chelatable free iron promotes tissue oxidation during reperfusion [75]. This notion is supported by the result where desferrioxamine, an iron chelator, has been shown to partially protect against ischemic lung injury induced by reperfusion [94]. Lung transplantation is also associated with iron accumulation in the lung and increased levels of transferrin and its receptor, lactoferrin and ferritin, while systemic iron levels are unchanged [95,96]. These findings further suggest metal-induced damage via oxidative stress in the lung and implies a potential benefit of local chelation therapy to deplete iron from the lung [96].

## Implications and Applications

It is now well recognized that the body has highly coordinated protective mechanisms from a variety of insults, such as pathogens, inflammation and oxidative stress by mobilizing or sequestering iron. Despite wide prevalence of lung injury and diseases that are associated with iron, there are few successful therapeutic advancements. Conventional methods to reduce iron burden in the body include dietary restriction, chelators and phlebotomy [38,97,98]. While still effective, these “passive or non-selective” interventions have considerable drawbacks such as systemic nutritional deficiency and severe adverse effects. These problems could be circumvented by “active or selective” therapies such as specific modulation of iron transporters, FPN and DMT1. For example, inhibitors of these transporters [99–101] might help decrease iron-associated cellular damage. Local or regional administration like inhalation of apotransferrin or other iron-chelating agents appear to be promising tools without perturbation of systemic iron homeostasis. Further study is clearly warranted to elucidate the benefits and risks of these potential therapeutic treatments in affecting changes in iron metabolism in lung injury and inflammation.

## Figures and Tables

**Table 1:** Characteristics of molecules that are involved in pulmonary iron homeostasis.

Molecules	Known or proposed functions	Lung cell type
Transferrin and its receptor	Import and export iron between airways or blood	Epithelial, Macrophage
Lactoferrin and its receptor	Import iron from the airway	Epithelial, Macrophage, Neutrophil
DMT1	Import iron into the cell	Epithelial, Macrophage
Nramp1	Import iron into the macrophage	Macrophage
Duodenal cytochrome b	Convert Fe^3+^ to Fe^2+^ at the apical membrane of epithelial cells	Epithelial
Ferritin	Store iron inside the cell; transport iron out of the cell	Epithelial, Macrophage
Siderocalin	Binds iron in a complex with endogenous catechol or siderophores secreted by invading pathogens	Epiethelial, Neutrophil
Hepcidin	Hormone regulating systemic iron metabolism that may exert local control in the lung	Epithelial
Ferroportin	Efflux intracellular iron to the airway	Epithelial, Macrophage

## Abbreviations

ARDS: Acute Respiratory Distress Syndrome
BAL: Bronchoalveolar Lavage
Dcytb: Duodenal cytochrome b
DMT1: Divalent Metal Transporter 1
FPN: Ferroportin
Lf: Lactoferrin
LfR: Lactoferrin Receptor
Nramp1: Natural Resistance-associated Macrophage Protein 1
PAP: Pulmonary Alveolar Proteinosis
RBC: Red Blood Cells
Tf: Transferrin
TfR: Transferrin Receptor
